# Relations between Objective and Perceived Built Environments and the Modifying Role of Individual Socioeconomic Position. A Cross-Sectional Study on Traffic Noise and Urban Green Space in a Large German City

**DOI:** 10.3390/ijerph15081562

**Published:** 2018-07-24

**Authors:** Steffen Andreas Schüle, Sarah Nanninga, Stefanie Dreger, Gabriele Bolte

**Affiliations:** 1 University of Bremen, Institute of Public Health and Nursing Research, Department of Social Epidemiology, 28359 Bremen, Germany; snanninga@uni-bremen.de (S.N.); stefanie.dreger@uni-bremen.de (S.D.); gabriele.bolte@uni-bremen.de (G.B.); 2 Health Sciences Bremen, University of Bremen, 28359 Bremen, Germany

**Keywords:** environmental inequalities, environmental health inequalities, vulnerability, built environment, noise, green space

## Abstract

Perceived annoyance due to traffic noise and lack of urban green space is mostly determined using data from self-administered questionnaires. However, there is still no clear evidence to what extent such perceived measures are related to objectively assessed environmental data and whether socioeconomic dimensions modify such relationships. In a cross-sectional study in Dortmund, Germany, georeferenced home addresses from parents with preschool aged children were used to analyse relations between exposures to objectively measured green space and traffic noise and subjective annoyance due to noise and lack of green space with the additional consideration of socioeconomic characteristics as effect modifiers. Higher perceived annoyance correlated with higher objectively measured traffic noise and lower objectively measured green, respectively. Stratified logistic regression models indicated a modifying role of socioeconomic characteristics. The strengths of associations between objectively measured environmental exposures and perceived annoyance differed by socioeconomic strata. Especially for noise, odds ratios were higher in low socioeconomic strata than in high socioeconomic strata. Therefore, using objective measures of the built environment as a proxy for individual perception should be made with caution as negative relations between objectively assessed built environments and health could be underestimated when considering individual socioeconomic position only as a confounder.

## 1. Introduction

Evidence from epidemiological studies shows that neighbourhood built environments are associated with individual health and health behaviours and contribute to social inequalities in health [1,2,3,4,5,6]. The built environment in urban areas covers many dimensions, such as transportation, land use, or public services which are both sources of environmental stressors (e.g., air and noise pollution) and environmental resources (e.g., urban green space and other recreational areas) [7].

To analyse environmental health risks and the contribution of environmental burdens and resources to environmental health inequalities, characteristics of the built environment can be measured either subjectively or objectively. Objective measures are mostly derived from land use plans, noise level data, or satellite images from remote sensing. Measures describing walkability [8,9], noise burdens [10,11], or distances to or availability of recreational areas or other facilities [12,13] are used as indicators to determine exposures to environmental burdens or resources of individuals. Another possibility to collect objective data of built environments are audit tools [14,15,16]. Subjective data about individually perceived environmental exposures are mostly collected with self-administered questionnaires [15,17,18].

There is mixed evidence on how objective and subjective measures of noise and green space are correlated. Moreover, both measures show different predictive powers for health outcomes and health behaviours. Studies on traffic noise exposure found out that objectively measured noise data and subjective noise annoyance showed weak to fair correlations [10,11,19,20,21,22,23]. Health related studies investigating both subjective and objective measures of noise exposures found out that objectively measured noise levels were more consistent in predicting cardiovascular health outcomes [10,20]. On the other hand, Nivison and Endresen found out that only perceived noise annoyance was associated with reported health complaints and sleeping problems, whereas objective noise levels showed no significant association [19].

For urban green space as an important environmental health resource there is also no clear evidence how objective and subjective measures are interrelated. Tilt et al. found out that objectively measured green space availability is a significant predictor for subjectively perceived availability [24]. However, there are also studies which found little agreement between subjective and objective measures of green space availability [25,26]. Besides that, studies showed that perceived green space availability was stronger related to individual use of green space and walking behaviours than objectively measured availability [24,27].

These mixed findings indicate that further research in this field is necessary. Especially when environmental health inequalities are analysed, the influence of socioeconomic characteristics on subjective environmental measures is relevant. There are two important conceptual pathways to consider when the development of environmental health inequalities is investigated: The first conceptual pathway hypothesizes a social unequal distribution of environmental exposures across socioeconomic groups. The second conceptual pathway hypothesizes different vulnerability across socioeconomic groups. This vulnerability model assumes that, given the same magnitude of environmental exposures, environmental adverse health effects are stronger in low socioeconomic groups compared to high socioeconomic groups [6,28]. In epidemiological studies socioeconomic vulnerability is mostly investigated with quantitative methods analysing effect modification, for example with stratification or interaction terms [29,30,31].

Assuming different vulnerability across socioeconomic groups this concept could also be very relevant when relationships between objective and subjective environmental measures are analysed. Different perceptions of the same objective environmental exposure across socioeconomic groups could result in a potential underestimation of environmental health inequalities if objective environmental measures are considered.

Most previous studies, however, which investigated relationships between subjective and objective built environmental characteristics and health did not consider dimensions of individual socioeconomic position (SEP), or included them only as an adjustment variable in multivariate analysis [10,11,22,24,25]. As a consequence, there is lack of evidence how objectively and subjectively assessed built environments and various dimensions describing individual socioeconomic disadvantage are interrelated. 

In this study the concept of different vulnerability across socioeconomic groups was applied. The main research question of this study was whether associations, firstly, between objectively measured data of traffic noise and subjectively measured noise annoyance and, secondly, between objectively measured green space availability and perceived lack of green space were modified by various dimensions of individual SEP. Our null hypothesis was that there was the same magnitude of association between objective and subjective environmental measures across socioeconomic groups. Our alternative hypothesis was that the magnitude of association between objective and subjective environmental measures was different across socioeconomic groups. Furthermore, overall relations between these objective and subjective environmental measures were analysed in order to contribute to the existing evidence on relations between objective and subjective environmental measures.

## 2. Materials and Methods

### 2.1. Study Area, Study Design, and Data Collection Procedure

Individual data were collected with self-administered parental questionnaires in a cross-sectional survey within the obligatory school entrance health examination for preschool aged children in the city of Dortmund, Germany, in 2015. Dortmund (280 km^2^) has about 600,000 inhabitants and is located in a highly urbanized region in the western part of Germany [32]. The local health authority gave the questionnaires to the parents during the health examination. The parents had the possibility to return the questionnaire directly to the health authority or to return it by post to the study centre.

The overall aim of the study was to investigate how perceived and objectively measured characteristics from the built and social neighbourhood environment were associated with physical and mental health outcomes in children. All parents gave their written consent. The study was approved by the ethics committee of the University of Bremen (Approval 06-3, 28.07.2014). Reported home addresses were geocoded with the Google Maps Geocoding Application Programming Interface [33]. Overall, 581 parents took part in the study and from 556 participants home addresses could be geocoded.

### 2.2. Objectively Measured Exposure to Traffic Noise

Based on a noise dispersion model, road traffic noise was modelled citywide in decibel (dB) at a 10 m point interval (for detailed information see [34]). The European standardized L_den_ (day-evening-night level) indicator was used which describes the average noise burden over 24 h with a stronger consideration of noise exposures in the evening and night hours (6:00 p.m.–10:00 p.m. and 10:00 p.m.–6:00 a.m.) [35]. The closest L_den_ point to each individual geocode was identified and used as a proxy for individual exposure to traffic noise.

The guidelines for community noise from the WHO recommend to use 55 dB as a threshold for noise exposures in outdoor environments because long term exposures >55 dB may have negative health consequences [36]. Based on these recommendations we created a binary variable of the assigned noise exposure levels to the individual home address applying a threshold of 55 dB.

### 2.3. Objectively Measured Green Space Availability

Green space availability was measured from two different data sources. Firstly, land use data from 2013 provided by the Regionalverband Ruhr containing land use categories at the parcel level were used [37]. These land use categories are conducted periodically mainly based on aerial photographs, automated land registration maps, and the official German basic map at the 1:5000 scale. Land use categories containing public green space (parks, botanical gardens, and zoos), green space close to multiple dwellings, and other green space including also public forests (deciduous, coniferous, and mixed forests) were extracted from the land use plan.

Secondly, urban greenness was determined with satellite images from remote sensing. Spectral bands from the Landsat 8 Operational Land Imager sensor [38] were downloaded with the Earth Explorer from the U.S. Geological Survey to calculate the Normalized Difference Vegetation Index (NDVI) [39]. A cloud free satellite image was used from the 21th of July 2013. The NDVI considers differences of visible and near-infrared wavelengths reflected on the earth surface because dense green vegetation absorbs and reflects these two wavelengths differently than sparse vegetation. The index has a range from −1 to +1 and values closer to +1 indicate more green whereas values closer to zero indicate less green [40].

A 400 m radius was drawn around each individual geocode to capture the close walkable neighbourhood environment. This distance was applied in many previous studies because it is approximately comparable to a 5 to 10 min walk [15,17,41,42]. Available green space in percent based on the selected land use categories and the average NDVI within the 400 m buffer were calculated for each geocode. All GIS based calculations were performed using ArcGIS 10.4 (Redlands, CA, USA).

### 2.4. Perceived Noise Annoyance and Lack of Green Space Availability

Parents were asked whether they felt annoyed by noise pollution and lack of green space in their close neighbourhood environment with the two following questions: “How strongly do you feel affected by noise in your neighbourhood?” and “How strongly do you feel affected by lack of accessible green space in your neighbourhood?”. Three categories of perceived annoyance (no, low vs. bearable vs. high, very high) were generated from a five-point Likert scale for comparing medians of objective measures across these three categories to analyse overall relations between objective and subjective environmental measures. These three categories were further reduced to binary variables (no, low vs. bearable, high, very high). These variables were modelled as dichotomous dependent outcome variables in stratified bivariate logistic regression models to analyse the modifying role of individual socioeconomic dimensions on associations between objective and subjective environmental measures.

### 2.5. Socioeconomic Characteristics of the Study Population

SEP of the study population was characterized as described before [43,44]. Briefly, three categories of parental education were defined. We considered the highest completed education achieved by either the father or the mother. The category ‘high’ included A-levels or advanced technical college entrance qualification. ‘Middle’ took into account upper secondary school certificate or adequate graduation. ‘Low’ included a lower secondary school certificate or no graduation.

The reported monthly household net income was considered as disposable income weighted for age and number of household members referring to the Organization for Economic Co-operation and Development-modified scale [45]. Sixty percent of the median household equivalent income in Germany based on the micro-census from 2014 was defined as the relative poverty threshold [46]. Three income categories were generated: ‘low’ (<60% of median), ‘middle’ (60% of median–median), and ‘high’ (>median).

Answers to four questions about single parenthood, family status, living together, and number of adult household members were combined to define a binary variable of single parenthood. Only consistent answers across these questions were considered.

Family unemployment was considered with a binary variable. Parental working status was defined as employed if one parent was employed for at least 15 h per week. Unemployment within households was assigned if both parents were marginally employed at most (less than 15 h per week). Migration background of the child was defined if both parents were born abroad or at least one parent and the child were born abroad or a different language than German was spoken at home [47].

### 2.6. Statistical Analysis

Firstly, to analyse overall relations between objective and subjective environmental measures without considering individual SEP, the Kruskall Wallis test was applied to compare the medians of objectively measured green space and noise exposures across the three categories of perceived noise annoyance and lack of green space. Data values were replaced by ranks and chi-square statistics were used to analyse differences in mean ranks. This non-parametric method was chosen because observations across perceived environmental categories were highly unbalanced (see Table 2) and the continuous response variables showed skewed distributions. However, as observations were all independent, approximately of the same form, and from a single population, assumptions of the Kruskal Wallis test were met [48]. The null hypothesis of the Kruskall Wallis test says that all samples are from the same distribution. A rejection provides evidence that at least one sample is from a distribution with a different location e.g., at least two medians differ [49,50]. In case of a significant Kruskall Wallis test, indicating significant differences between at least two medians, the Dwass, Steel, Critchlow-Fligner post-hoc test for multiple comparison was applied in order to analyse which pairs of categories were significantly different from each other [51].

Secondly, to analyse the modifying role of individual socioeconomic dimensions on associations between objective and subjective environmental measures bivariate logistic regression models were calculated and stratified by socioeconomic dimensions. Binary variables of perceived annoyance were modelled as dichotomous dependent outcome variables and objective measures of noise and green space availability were considered as independent variables. 

In all logistic regression models traffic noise was modelled as a binary variable with the threshold of 55 dB. NDVI values and percentages of green space were modelled as continuous variables. All statistical analyses were performed using SAS statistical software package version 9.4 (SAS Institute, Cary, NC, USA).

## 3. Results

### 3.1. Characteristics of the Study Population

Overall, 36.0% of the study population were exposed to traffic noise ≥55 dB. The respondents had on average 9.8% available green space and a mean NDVI of 0.33 within a 400 m radius around their home address (see Table 1). 11.5% of the parents felt highly or very highly annoyed due to noise exposure and 7.2% by lack of green space in their neighbourhood environment (see Table 2).

### 3.2. Relationships between Objectively Measured Exposures and Perceived Environmental Annoyance

A higher perceived noise annoyance was related to higher median values of objectively measured traffic noise. Furthermore, a higher annoyance due to lack of green space in the living environment corresponded to a lower percentage of green space and lower median NDVI values. The *p*-values of the Kruskall Wallis test showed that at least one pair of medians was significantly different (see Table 3).

Multiple comparison tests between single pairs of median values are given in Table 4. There was a significant difference in medians between the lowest and highest pair of annoyance category across all objective environmental measures. For the other median pairs *p*-values did not show a consistent pattern and both significant and non-significant differences were found.

### 3.3. Effect Modification by SEP

Logistic regression models stratified by socioeconomic dimensions confirmed the overall relationships from the Kruskall Wallis test that objectively measured exposures in terms of lack of green space and high traffic noise were associated with perceived annoyance (see Table 5). Stratified models indicated a modifying role of individual SEP on associations between objective measures and perceived annoyance. Although there were often wide and non-significant confidence intervals in the single strata, there was a detectable trend, especially for traffic noise, that the magnitude of associations between objective and perceived environmental measures differed by single socioeconomic strata.

Odds ratios which showed overall positive associations between exposure to objective traffic noise and perceived noise annoyance were higher for individuals with a low education, living in low income and unemployed households, or being a single parent. For example, parents from the unemployed households stratum being exposed to objectively measured noise ≥55 dB had a 7.5 higher chance to feel annoyed by noise compared to parents of this stratum being exposed to noise <55 dB (reference category). In contrast, parents being exposed to the same objectively measured noise exposure of ≥55 dB from the employed households stratum had only a 2.22 higher chance to feel annoyed by noise compared to parents of this stratum being exposed to noise <55 dB (reference category). For migration background differences in the odds ratios were marginal.

There was some indication of effect modification by SEP on associations between NDVI, percentage of green space, and perceived annoyance due to lack of green space. However, the pattern of higher annoyances in people with low SEP was not consistent across the SEP dimensions.

## 4. Discussion

Our study showed that perceived annoyance due to noise and lack of green was related to a higher objectively measured environmental burden of traffic noise and lack of green space. Further, our study indicated that SEP modified the relationship between objectively measured noise and perceived noise annoyance. People with a low SEP had a higher chance feeling annoyed by the same magnitude of the objectively measured environmental noise exposure than people with a high SEP. There was no consistent trend of a modifying role of SEP on the pathway between objective measures of green space and perceived annoyance due to lack of green.

Previous studies found correlations between objective environmental exposures and perceived annoyance or availability which are in line with our result of median differences of objective noise and green measures across categories of perceived annoyance. Three studies, which calculated Spearman rank correlation coefficients, found correlations between categories of perceived traffic annoyance and objectively measured traffic noise levels [10,22,52]. A study by Björk et al. compared percentages of people feeling annoyed by road traffic noise across three exposure categories based on road noise levels. A significant higher noise annoyance was detected with higher objective exposure levels [11]. NDVI measures around home addresses showed also positive correlations with perceived amount of natural outdoor environments in a study conducted in Barcelona [53].

Our multiple comparison tests showed that not all median values were significantly different from each other. Previous studies which focused more on agreement instead of correlation indicated that subjective and objective measures of green space availability may reflect partially different constructs [25,26]. De Jong et al. found correlations between the availability of five subjectively and objectively assessed characteristics of green space around individual home addresses, such as spaciousness, quietness, or lushness. However, when agreement was measured between subjective and objective availability of these characteristics with Cohen’s kappa statistic low agreement was found [26]. A study by Leslie et al. also applied Cohen’s kappa statistic to analyse agreement between subjective green space availability and objectively measured NDVI values in the close living environment. They found little agreement between various dimensions of perceived green space characteristics derived from factor analysis and the objectively measured NDVI [25]. 

Agreement and correlation have different concepts resulting in different statistics, such as Spearman or Pearson correlation coefficients for analysing correlation or Cohen’s kappa for analysing agreement [54]. In contrast to correlation, agreement assumes that the same construct is measured, such as the diagnosis of a disease or the assumption of assessing environments as noisy vs. not noisy or green vs. not green. Therefore, analysis of agreement requires a clear definition of ‘present’ or ‘absent’ of the outcome in order to measure the degree of concordance between individuals. For environmental outcomes, especially for green space, there is still a great heterogeneity and an ongoing discussion in the scientific literature how green space could be defined and measured in studies on environment and health [55]. Therefore, there is still no gold standard which cut-off should be used to define a green environment as ‘present’ or ‘absent’ which makes comparisons of results across studies challenging.

Other studies predicting noise annoyance or satisfaction with green space with multivariate regression techniques found independent positive associations between objectively assessed environments and subjective measures [21,24]. Higher averages from estimated 24 h day-night levels of traffic noise in Windsor, Canada, were significantly associated with a higher subjective noise annoyance independent of sex, age, and area deprivation [21]. A study by Tilt et al. measured green space availability with the NDVI based on parcels in Seattle with a 0.4 mile walking distance around them. Subjective green space availability was measured with an index capturing availability of eight dimensions of natural characteristics, such as trees, wildlife, or natural vegetation around the home address. In linear regression, NDVI was significantly positively associated with subjective greenness independent of sociodemographic characteristics [24].

However, many studies further found out that objective environmental measures do not adequately predict subjective annoyance and further characteristics are important to consider, such as sociodemographic or psychosocial factors [14,17,23,56]. As pointed out in a conceptual model by Riedel et al. [23] there could be additional factors on the pathway between objectively measured noise exposures and the subjective response to noise, such as factors of neighbourhood satisfaction, socioeconomic and demographic factors, health-related factors, or attitudinal factors. 

A study by Mackenbach et al. [14] analysed the contribution of both objective built environments and further social and health related characteristics to differences in perceived environments between neighbourhoods in five urban regions of Europe. Neighbourhood perception was operationalized with an index capturing safety, aesthetics, functionality, and destinations. Results from multilevel regression analysis showed that objective measures of traffic, aesthetics, functionality, and destinations explained only 15% of differences of perceived environments between neighbourhoods. Social cohesion explained most between neighbourhood variance of perceived environments, followed by further individual characteristics, such as physical activity or self-rated health. 

A systematic review by Orstad et al. found out that apart from subjective and objective environmental measures, measures describing marital status or SEP, especially income, explained agreement between subjective and objective physical activity environments [56]. A study from Wisconsin also showed that education was a significant predictor for discordance between perceived and objectively measured availability of various neighbourhood destinations, such as parks, trails, or stores [17].

Most of these studies identified only independent predictors for subjective environmental annoyance and did not consider effect modification. Thus, there is still lack of knowledge to what extent various socioeconomic factors modify relationships between objectively measured environments and perceived environmental annoyance in terms of socioeconomic vulnerability. Our study indicated that under the same magnitude of the objectively measured environmental exposure individuals with a low SEP might have a higher chance to perceive this exposure as more annoying than people with a high SEP. This was consistent for noise.

Duration of stay at home could explain the strong effect modification for single parents or unemployed people as they might spend more time at home and in their neighbourhood environment and are therefore longer exposed to environmental exposures in their immediate living environment. This explanation is supported by findings from a study conducted in Belgrade which found a positive association between the time spent at home and subjective traffic noise annoyance [57].

The concept of allostatic load provides further entry points for explaining overall differences in effect modification by SEP on the pathway between objective and perceived environmental exposures. Allostatic load refers to the physiologic response to perceived and chronic individual stress [58]. Central parts of this concept are social, psychological, and environmental stressors on the small-area and individual level influencing individual stress levels [59,60]. Studies from this research field suggest that a low SEP result in a faster accumulation of biological “wear and tear”, defined as allostatic load [61]. Potential interactions between psychosocial and environmental processes and the amount of multiple stressors being exposed to could explain increased vulnerability of low SEP individuals.

### 4.1. Limitations

One limitation of this study is that our study population consisted of parents only. Based on that, our results may not be generalized to the overall adult population in Germany. A second limitation are the unequal sample sizes across groups of perceived annoyance categories and the skewed data distributions resulting in the application of non-parametric statistics which are less powerful. Thirdly, odds ratios from stratified logistic regression should be interpreted with caution as confidence intervals were very wide which was mainly due to the small sample size. Further studies are necessary including a representative adult population sample with a higher sample size in order to adequately analyse moderating effects of socioeconomic dimensions.

### 4.2. Strengths

Firstly, one strength of this study was that different objective measures for urban green were used: the NDVI from remote sensing which considered all kinds of public and private green, and data from land use categories containing mainly public green space. Secondly, this study considered various dimensions of individual SEP in order to consider their potentially different vulnerability effects. Thirdly, all environmental exposures were assigned on the individual level using home addresses.

## 5. Conclusions

Against the background of the evidence available so far, we recommend to use subjective and objective environmental measures interchangeably with caution, especially when focusing on environmental health inequalities where moderating effects of socioeconomic dimensions on subjective environmental annoyance should be considered. Assuming that perception of the environment is relevant for predicting health outcomes the use of objective environmental measures may lead to underestimation of health impacts when socioeconomic dimensions are only taken into account as confounders. Individuals with a low SEP could perceive the same magnitude of objectively measured environmental exposures more badly than individuals with high SEP resulting in a potential underestimation of existing environmental health inequalities if only objective environmental measures are analysed.

There is further need for research on explaining underlying constructs of vulnerability in the context of environmental stressors and resources. There is still lack of evidence regarding the quantification of interactions between individual psychosocial characteristics and different environmental exposures as one important contributor to socioeconomic vulnerability. Besides that, studies in the future should be aware of potential different underlying mechanisms of various socioeconomic dimensions, such as unemployment, occupation, education, or income, explaining increased vulnerability of people with a low SEP to environmental burdens and lack of environmental resources.

## Figures and Tables

**Table 1:** Characteristics of exposures to objectively measured built environments.

	*N*	Mean	Median	Std. Dev.	Min	Max
Traffic noise continuously [dB]	556	53.32	52.58	7.57	37.86	73.73
Traffic noise binary [dB]						
≥55 dB	200					
<55 dB	356					
Green space [%] ^1^	556	9.80	7.30	8.45	0	56.95
NDVI [value range: −1 to +1] ^1^	556	0.33	0.34	0.06	0.11	0.47

*N* = total number of observations; NDVI = Normalized Difference Vegetation Index; ^1^ including a 400 m radius around individual geocode.

**Table 2:** Characteristics of the study population.

Socioeconomic Dimensions	*N*	Percentage
Parental education		
Low	34	6.14
Middle	96	17.33
High	424	76.53
Equivalent household income		
Low (<60% of median income ^1^)	74	13.78
Middle (60% to median income)	157	29.24
High (>median income)	306	56.98
Parental working status		
Unemployment within household	43	7.75
At least one parent employed	512	92.25
Single parenthood		
Single parent	68	12.39
Other	481	87.61
Migration background of the child		
Yes	135	24.32
No	420	75.68
**Perceived Annoyance due to Built Environments**		
Exposure to noise burden		
High/very high	64	11.51
Bearable	107	19.24
No/small	385	69.24
Lack of green space in neighbourhood		
High/very high	40	7.19
Bearable	63	11.33
No/small	453	81.47

*N* = total number of observations; ^1^ Median equivalent household income in Germany.

**Table 3:** Median values of objectively measured environmental exposures to green space and traffic noise across categories of perceived annoyance.

Objectively Measured Exposures	No/Small Annoyance	Bearable Annoyance	High/Very High Annoyance	*p*-Values of Kruskall Wallis Test
Traffic noise [dB]	51.7 (*n* = 385)	54.40 (*n* = 107)	57.65 (*n* = 64)	<0.05
Green space [%] ^1^	7.54 (*n* = 453)	7.34 (*n* = 63)	4.72 (*n* = 40)	<0.05
NDVI [value range: −1 to +1] ^1^	0.34 (*n* = 453)	0.31 (*n* = 63)	0.28 (*n* = 40)	<0.05

*n* = total number of observations; NDVI = Normalized Difference Vegetation Index; ^1^ including a 400 m radius around individual geocode.

**Table 4:** Results of multiple comparison test for single pairs of medians.

Pairwise Comparison of Annoyance Categories	Traffic Noise [dB]	Green Space [%] ^1^	NDVI [Value Range: −1 to +1] ^1^
No/small vs. bearable	<0.01 ^2^	0.97	<0.05
No/small vs. high/very high	<0.01	<0.05	<0.05
Bearable vs. high/very high	0.18	<0.05	0.52

NDVI = Normalized Difference Vegetation Index; ^1^ including a 400 m radius around individual geocode; ^2^ *p*-values from Dwass, Steel, Critchlow-Fligner test.

**Table 5:** Modification of the association between objectively measured built environments and perceived annoyance by socioeconomic characteristics.

Socioeconomic Strata	Traffic Noise ≥55 dB (Reference: <55 dB)	NDVI, per 0.1 Unit (Value Range: −1 to +1) ^1^	Green Space, per 10% ^1^
Parental education			
Low	3.28 (0.73–14.68) ^2^	0.42 (0.14–1.28)	0.75 (0.22–2.53)
Middle	2.63 (1.14–6.06)	0.13 (0.05–0.38)	0.32 (0.12–0.88)
High	2.35 (1.52–3.65)	0.27 (0.17–0.42)	0.72 (0.50–1.04)
Equivalent household income			
Low (<60% of median income ^3^)	3.12 (1.18–8.22)	0.41 (0.18–0.90)	0.56 (0.24–1.30)
Middle (60% to median income)	2.76 (1.42–5.37)	0.14 (0.07–0.31)	0.56 (0.31–1.02)
High (>median income)	2.10 (1.22–3.60)	0.31 (0.18–0.55)	0.66 (0.39–1.11)
Parental working status			
Unemployment within household	7.50 (1.87–30.16)	0.19 (0.05–0.68)	0.66 (0.21–2.07)
At least one parent employed	2.22 (1.50–3.28)	0.27 (0.18–0.40)	0.63 (0.44–0.90)
Single parenthood			
Single parent	5.23 (1.72–15.87)	0.23 (0.10–0.58)	0.65 (0.28–1.51)
Other	2.24 (1.50–3.35)	0.26 (0.17–0.40)	0.64 (0.44–0.92)
Migration background of the child			
Yes	2.16 (1.04–4.47)	0.30 (0.15–0.58)	1.00 (0.99–1.00)
No	2.64 (1.71–4.07)	0.24 (0.15–0.37)	0.99 (0.99–1.00)

NDVI = Normalized Difference Vegetation Index; ^1^ including a 400 m radius around individual geocode; ^2^ Odds ratios and 95% confidence intervals from bivariate logistic regression; ^3^ Median equivalent household income in Germany.

## References

[1] Schüle S.A., Bolte G. (2015). Interactive and independent associations between the socioeconomic and objective built environment on the neighbourhood level and individual health: A systematic review of multilevel studies. PLoS ONE.

[2] Ding D., Gebel K. (2012). Built environment, physical activity, and obesity: What have we learned from reviewing the literature?. Health Place.

[3] Gong Y., Palmer S., Gallacher J., Marsden T., Fone D. (2016). A systematic review of the relationship between objective measurements of the urban environment and psychological distress. Environ. Int..

[4] Bancroft C., Joshi S., Rundle A., Hutson M., Chong C., Weiss C.C., Genkinger J., Neckerman K., Lovasi G. (2015). Association of proximity and density of parks and objectively measured physical activity in the United States: A systematic review. Soc. Sci. Med..

[5] Cummins S.K., Jackson R.J. (2001). The built environment and children’s health. Pediatr. Clin..

[6] WHO (2012). Environmental Health Inequalities in Europe.

[7] Northridge M.E., Sclar E.D., Biswas P. (2003). Sorting out the connections between the built environment and health: A conceptual framework for navigating pathways and planning healthy cities. J. Urban Health.

[8] Hanibuchi T., Nakaya T., Yonejima M., Honjo K. (2015). Perceived and Objective Measures of Neighborhood Walkability and Physical Activity among Adults in Japan: A Multilevel Analysis of a Nationally Representative Sample. Int. J. Environ. Res. Public Health.

[9] Frank L.D., Sallis J.F., Saelens B.E., Leary L., Cain K., Conway T.L., Hess P.M. (2010). The development of a walkability index: Application to the Neighborhood Quality of Life Study. Br. J. Sports Med..

[10] Babisch W., Pershagen G., Selander J., Houthuijs D., Breugelmans O., Cadum E., Vigna-Taglianti F., Katsouyanni K., Haralabidis A.S., Dimakopoulou K. (2013). Noise annoyance—A modifier of the association between noise level and cardiovascular health?. Sci. Total Environ..

[11] Björk J., Ardö J., Stroh E., Lövkvist H., Östergren P.-O., Albin M. (2006). Road traffic noise in southern Sweden and its relation to annoyance, disturbance of daily activities and health. Scand. J. Work Environ. Health.

[12] Gascon M., Triguero-Mas M., Martinez D., Dadvand P., Forns J., Plasencia A., Nieuwenhuijsen M.J. (2015). Mental health benefits of long-term exposure to residential green and blue spaces: A systematic review. Int. J. Environ. Res. Public Health.

[13] Lee S.M., Conway T.L., Frank L.D., Saelens B.E., Cain K.L., Sallis J.F. (2016). The Relation of Perceived and Objective Environment Attributes to Neighborhood Satisfaction. Environ. Behav..

[14] Mackenbach J.D., Lakerveld J., van Lenthe F.J., Bardos H., Glonti K., Compernolle S., De Bourdeaudhuij I., Oppert J.M., Roda C., Rutter H. (2016). Exploring why residents of socioeconomically deprived neighbourhoods have less favourable perceptions of their neighbourhood environment than residents of wealthy neighbourhoods. Obes. Rev..

[15] Boehmer T.K., Hoehner C.M., Deshpande A.D., Brennan Ramirez L.K., Brownson R.C. (2007). Perceived and observed neighborhood indicators of obesity among urban adults. Int. J. Obes..

[16] Kamphuis C.B., Mackenbach J.P., Giskes K., Huisman M., Brug J., van Lenthe F.J. (2010). Why do poor people perceive poor neighbourhoods? The role of objective neighbourhood features and psychosocial factors. Health Place.

[17] Bailey E.J., Malecki K.C., Engelman C.D., Walsh M.C., Bersch A.J., Martinez-Donate A.P., Peppard P.E., Nieto F.J. (2014). Predictors of discordance between perceived and objective neighborhood data. Ann. Epidemiol..

[18] Wen M., Hawkley L.C., Cacioppo J.T. (2006). Objective and perceived neighborhood environment, individual SES and psychosocial factors, and self-rated health: An analysis of older adults in Cook County, Illinois. Soc. Sci. Med..

[19] Nivison M.E., Endresen I.M. (1993). An Analysis of Relationships among Environmental Noise, Annoyance and Sensitivity to Noise, and the Consequences for Health and Sleep. J. Behav. Med..

[20] Pitchika A., Hampel R., Wolf K., Kraus U., Cyrys J., Babisch W., Peters A., Schneider A. (2017). Long-term associations of modeled and self-reported measures of exposure to air pollution and noise at residence on prevalent hypertension and blood pressure. Sci. Total Environ..

[21] Oiamo T.H., Baxter J., Grgicak-Mannion A., Xu X., Luginaah I.N. (2015). Place effects on noise annoyance: Cumulative exposures, odour annoyance and noise sensitivity as mediators of environmental context. Atmos. Environ..

[22] Birk M., Ivina O., von Klot S., Babisch W., Heinrich J. (2011). Road traffic noise: Self-reported noise annoyance versus GIS modelled road traffic noise exposure. J. Environ. Monit..

[23] Riedel N., Scheiner J., Müller G., Köckler H. (2013). Assessing the relationship between objective and subjective indicators of residential exposure to road traffic noise in the context of environmental justice. J. Environ. Plan. Manag..

[24] Tilt J.H., Unfried T.M., Roca B. (2007). Using objective and subjective measures of neighborhood greenness and accessible destinations for understanding walking trips and BMI in Seattle, Washington. Am. J. Health Promot..

[25] Leslie E., Sugiyama T., Ierodiaconou D., Kremer P. (2010). Perceived and objectively measured greenness of neighbourhoods: Are they measuring the same thing?. Landsc. Urban Plan..

[26] De Jong K., Albin M., Skaback E., Grahn P., Wadbro J., Merlo J., Bjork J. (2011). Area-aggregated assessments of perceived environmental attributes may overcome single-source bias in studies of green environments and health: Results from a cross-sectional survey in Southern Sweden. Environ Health.

[27] Wang D., Brown G., Liu Y., Mateo-Babiano I. (2015). A comparison of perceived and geographic access to predict urban park use. Cities.

[28] Bolte G., Pauli A., Hornberg C., Jerome O.N. (2011). Environmental justice: Social disparities in environmental exposures and health: Overview. Encyclopedia of Environmental Health.

[29] Bell M.L., Zanobetti A., Dominici F. (2013). Evidence on vulnerability and susceptibility to health risks associated with short-term exposure to particulate matter: A systematic review and meta-analysis. Am. J. Epidemiol..

[30] Dragano N., Hoffmann B., Moebus S., Moehlenkamp S., Stang A., Verde P.E., Jockel K.H., Erbel R., Siegrist J., Heinz Nixdorf Recall Study Investigative Group (2009). Traffic exposure and subclinical cardiovascular disease: Is the association modified by socioeconomic characteristics of individuals and neighbourhoods? Results from a multilevel study in an urban region. Occup. Environ. Med..

[31] Cakmak S., Hebbern C., Cakmak J.D., Vanos J. (2016). The modifying effect of socioeconomic status on the relationship between traffic, air pollution and respiratory health in elementary schoolchildren. J. Environ. Manag..

[32] Stadt Dortmund—Dortmunderstatistik Dortmund Auf Einen Blick. https://www.dortmund.de/media/p/statistik_3/statistik/00_01_Eckdaten.pdf.

[33] Google Google Maps Geocoding Application Programming Interface. https://developers.google.com/maps/documentation/geocoding/intro?hl=en.

[34] Ministerium für Umwelt Landwirtschaft Natur- und Verbraucherschutz des Landes Nordrhein-Westfalen Umgebungslärm in NRW. http://www.umgebungslaerm.nrw.de/.

[35] European Environment Agency (2014). Noise in Europe 2014.

[36] Berglund B., Lindvall T., Schwela D.H., WHO (1999). Guidelines for Community Noise.

[37] Regionalverband Ruhr: Daten für die Stadt- und Regionalplanung. https://www.metropoleruhr.de/regionalverband-ruhr/karten-geodaten/geodaten/flaechennutzungskartierung.html.

[38] Roy D.P., Wulder M.A., Loveland T.R., Woodcock C.E., Allen R.G., Anderson M.C., Helder D., Irons J.R., Johnson D.M., Kennedy R. (2014). Landsat-8: Science and product vision for terrestrial global change research. Remote Sens. Environ..

[39] U.S. Geological Survey Earth Explorer. https://earthexplorer.usgs.gov/.

[40] Weier J., Herring D. Measuring Vegetation (NDVI & EVI). https://earthobservatory.nasa.gov/Features/MeasuringVegetation/.

[41] Hoehner C.M., Brennan Ramirez L.K., Elliott M.B., Handy S.L., Brownson R.C. (2005). Perceived and objective environmental measures and physical activity among urban adults. Am. J. Prev. Med..

[42] Pikora T.J., Bull F.C., Jamrozik K., Knuiman M., Giles-Corti B., Donovan R.J. (2002). Developing a reliable audit instrument to measure the physical environment for physical activity. Am. J. Prev. Med..

[43] Bolte G., Fromme H., for the GME Study Group (2009). Socioeconomic determinants of children’s environmental tobacco smoke exposure and family’s home smoking policy. Eur. J. Public Health.

[44] Scharte M., Bolte G., for the GME Study Group (2013). Increased health risks of children with single mothers: The impact of socio-economic and environmental factors. Eur. J. Public Health.

[45] Gallie D., Paugam S. (2003). Social Precarity and Social Integration.

[46] Statistisches Bundesamt (Destatis) Armutsgefährdungsschwelle. https://www.destatis.de/DE/ZahlenFakten/GesellschaftStaat/Soziales/Sozialberichterstattung/Tabellen/Armutsgefaehrungsschwelle.html.

[47] Klingshirn H., Hendrowarsito L., Fromme H., Bolte G., for the GME Study Group (2014). The impact of migration background on children’s secondhand smoke exposure. A cross-sectional study within the health monitoring units (GME) in Bavaria, Germany. Gesundheitswesen.

[48] Kruskal W.H., Wallis W.A. (1952). Use of Ranks in One-Criterion Variance Analysis. J. Am. Stat. Assoc..

[49] Gibbons J.D., Chakraborti S. (2011). Nonparametric Statistical Inference.

[50] Sprent P. (1989). Applied Nonparametric Statistical Methods.

[51] Hollander M., Wolfe D.A. (1999). Nonparametric Statistical Methods.

[52] Von Szombathely M., Albrecht M., Augustin J., Bechtel B., Dwinger I., Gaffron P., Krefis A., Oßenbrügge J., Strüver A. (2018). Relation between Observed and Perceived Traffic Noise and Socio-Economic Status in Urban Blocks of Different Characteristics. Urban Sci..

[53] Zijlema W.L., Triguero-Mas M., Smith G., Cirach M., Martinez D., Dadvand P., Gascon M., Jones M., Gidlow C., Hurst G. (2017). The relationship between natural outdoor environments and cognitive functioning and its mediators. Environ. Res..

[54] Liu J., Tang W., Chen G., Lu Y., Feng C., Tu X.M. (2016). Correlation and agreement: Overview and clarification of competing concepts and measures. Shanghai Arch. Psychiatry.

[55] WHO (2016). Urban Green Spaces and Health.

[56] Orstad S.L., McDonough M.H., Stapleton S., Altincekic C., Troped P.J. (2016). A Systematic Review of Agreement Between Perceived and Objective Neighborhood Environment Measures and Associations with Physical Activity Outcomes. Environ. Behav..

[57] Paunovic K., Jakovljevic B., Belojevic G. (2009). Predictors of noise annoyance in noisy and quiet urban streets. Sci. Total Environ..

[58] McEwen B.S., Stellar E. (1993). Stress and the individual. Mechanisms leading to disease. Arch. Intern. Med..

[59] Beckie T.M. (2012). A systematic review of allostatic load, health, and health disparities. Biol. Res. Nurs..

[60] Morello-Frosch R., Shenassa E.D. (2006). The environmental “Riskscape” and social inequality: Implications for explaining maternal and child health disparities. Environ. Health Perspect..

[61] Seeman T., Epel E., Gruenewald T., Karlamangla A., McEwen B.S. (2010). Socio-economic differentials in peripheral biology: Cumulative allostatic load. Ann. N. Y. Acad. Sci..

